# Development and mapping of DArT markers within the *Festuca - Lolium *complex

**DOI:** 10.1186/1471-2164-10-473

**Published:** 2009-10-15

**Authors:** David Kopecký, Jan Bartoš, Adam J Lukaszewski, James H Baird, Vladimír Černoch, Roland Kölliker, Odd Arne Rognli, Helene Blois, Vanessa Caig, Thomas Lübberstedt, Bruno Studer, Paul Shaw, Jaroslav Doležel, Andrzej Kilian

**Affiliations:** 1 Laboratory of Molecular Cytogenetics and Cytometry, Institute of Experimental Botany, Sokolovská 6, CZ-77200, Olomouc, Czech Republic; 2 Department of Botany and Plant Sciences, University of California, Riverside, CA 92521, USA; 3 Šlechtitelská stanice Hladké Životice, s.r.o., Fulnecká 95, Hladké Životice, CZ-74247, Czech Republic; 4 Agroscope Reckenholz Tanikon Research Station ART, Reckenholzstr 191, CH-8046 Zurich, Switzerland; 5 Department of Plant and Environmental Sciences, Norwegian University of Life Sciences, PO Box 5003, N-1432, Aas, Norway; 6 Diversity Arrays Technology, 1 Wilf Crane Crescent, Yarralumla, ACT 2600, Australia; 7 Iowa State University, Agronomy Department, 1204 Agronomy Hall, Ames, IA 50011, USA; 8 University of Aarhus, Department of Genetics and Biotechnology, Research Centre Flakkebjerg, DK-4200 Slagelse, Denmark; 9 Plant Bioinformatics Group, Scottish Crop Research Institute, Invergowrie, Dundee, DD2 5DA, UK

## Abstract

**Background:**

Grasses are among the most important and widely cultivated plants on Earth. They provide high quality fodder for livestock, are used for turf and amenity purposes, and play a fundamental role in environment protection. Among cultivated grasses, species within the *Festuca-Lolium *complex predominate, especially in temperate regions. To facilitate high-throughput genome profiling and genetic mapping within the complex, we have developed a Diversity Arrays Technology (DArT) array for five grass species: *F. pratensis*, *F. arundinacea*, *F. glaucescens*, *L. perenne *and *L. multiflorum*.

**Results:**

The DArTFest array contains 7680 probes derived from methyl-filtered genomic representations. In a first marker discovery experiment performed on 40 genotypes from each species (with the exception of *F. glaucescens *for which only 7 genotypes were used), we identified 3884 polymorphic markers. The number of DArT markers identified in every single genotype varied from 821 to 1852. To test the usefulness of DArTFest array for physical mapping, DArT markers were assigned to each of the seven chromosomes of *F. pratensis *using single chromosome substitution lines while recombinants of *F. pratensis *chromosome 3 were used to allocate the markers to seven chromosome bins.

**Conclusion:**

The resources developed in this project will facilitate the development of genetic maps in *Festuca *and *Lolium*, the analysis on genetic diversity, and the monitoring of the genomic constitution of the *Festuca × Lolium *hybrids. They will also enable marker-assisted selection for multiple traits or for specific genome regions.

## Background

Grasses are among the most important and widely cultivated plants on Earth, with a total area of grassland estimated to be twice that of cropland. In Europe, fifty percent of the farmed landscape is under grasses which accounts for a large proportion of the annual production of beef and milk with a total value of more than € 70 billion [1]. They are also used extensively for turf and amenity purposes, and play an important role in soil conservation and protection of environmental resources. Among the cultivated grasses, ryegrasses (*Lolium *spp.) and fescues (*Festuca *spp.) predominate, especially in temperate climates [2].

Fescues and ryegrasses are closely related and belong to the *Poaceae*. Whereas *Lolium *consists of only eight diploid species, *Festuca *comprises nearly 500 species with ploidy levels ranging from diploid to dodecaploid. The two agronomically exploited fescues - *F. pratensis *and *F. arundinacea *- belong to the *Bovinae *section, subgenus *Schedonorus *[3,4], but the systematics of *Lolium *and *Festuca *are still a subject for review. Darbyshire [5] proposed to merge *Festuca *subgenus *Schedonorus *with the *Lolium*. However, clear differentiation of *Lolium *and *Festuca *into two separate taxa is supported based on morphological traits, isozymes [6], RAPD markers [7], analysis of ITS and the chloroplast *trn *region [8], as well as EST-SSR markers [9].

Decades of breeding resulted in superior ryegrass and fescue cultivars outperforming their wild progenitors. However, there is a risk that some desirable alleles of the progenitors were lost during the breeding process. The analysis of genetic diversity in cultivars of *Dactylis glomerata *and *Gossypium arboreum *revealed lower levels of intraspecific variability as compared to the wild types [10,11]. Similar trends were observed in *F. pratensis *[7] and *L. perenne *[12]. Investigations of genetic diversity of Nordic *F. pratensis *cultivars and natural populations have shown that although the molecular genetic variation has not been reduced by breeding [13], the phenotypic variation in important agronomic traits is lower in cultivars when compared to natural populations [14]. Thus, the existing gene pool of wild accessions represents an invaluable source of alleles, which can be introduced into existing cultivars. The risk of erosion of species' gene pools calls for characterization of the natural genetic variability and its conservation in gene banks. An ideal system to describe genetic diversity should allow for parallel screening of thousands of genomic loci and quickly analyze many accessions at a low cost. Such a method will allow more effective selection of material for breeding purposes.

A high-throughput genotyping system is also required to speed up the development of new cultivars with desirable attributes using marker assisted selection (MAS). For MAS, the development of genetic markers and genetic linkage maps is required. Several genetic maps were constructed for *Lolium *spp. using isozymes, RFLP, AFLP, RAPD, SSR, and EST-derived CAPS markers [15-21]. Markers associated with traits such as disease resistance [22-24], winter hardiness [25], quality parameters [26] and fertility traits [27] have been identified through the analysis of quantitative trait loci (QTL). Within fescue species, genetic maps were generated for both agronomically important species - *F. arundinacea *and *F. pratensis *[28,29]. The identification of QTLs for resistance to biotic and abiotic stresses as well as the application of genetic markers in cultivar development has been recently summarized by Zhang et al. [30].

In addition to breeding of fescue and ryegrass cultivars, some breeding programs have released cultivars originating from intergeneric crosses of *Festuca *with *Lolium *(called Festuloliums), thereby combining desirable agronomic characteristics of both genera [2]. The ryegrass species - *L. multiflorum *Lam. and *L. perenne *L. are highly nutritious, palatable and digestible, and display numerous valuable characteristics for turf culture including deep green color, uniformity, and rapid establishment. Fescues are known for their adaptation to abiotic stresses. In particular, *F. arundinacea *is well known for its drought tolerance, while *F. pratensis *carries genes for winter hardiness. Until recently, the genomic constitution of commercial Festulolium cultivars was mostly a matter of speculation and extrapolation from research stocks. Kopecký et al. [31], using genomic *in situ *hybridization (GISH), demonstrated that genomic composition of the commercial Festulolium varied significantly, depending on the parents used for hybrid development and the breeding strategy employed. While GISH is a very powerful and simple method for karyotypic studies in the *Lolium-Festuca *complex, it does have limited resolution. While this resolution limit has not yet been established in grasses, in wheat-rye hybrids it was found to be up to the Mb level [32]. Obviously, this limits the technique to identification of large genomic regions. Low throughput is another bottleneck preventing its broader use in breeding. Thus, a more sensitive and effective system is needed for genotyping in breeding of commercial Festulolium cultivars.

The need for a high-throughput genotyping system led to the development of various DNA arrays and chips (reviewed in [33]). Although they are based on different principles, all of them can be used to screen thousands or hundreds of thousands genomic loci in a single pass. However, most of them are based on scoring single nucleotide or single feature polymorphisms (SNP and SFP, respectively) and the development of such markers requires detailed knowledge of the DNA sequence. Therefore, it can only be used with some degree of success in species with at least some minimum level of DNA sequencing completed. In contrast, Diversity Arrays Technology (DArT) is a microarray hybridization based technique that permits simultaneous screening thousands of polymorphic loci without any prior sequence information [34]. DArT is high-throughput, low-cost, quick and reproducible. It has been used for genotyping and genetic mapping of genomes in numerous species, such as *Arabidopsis thaliana *[35], *Hordeum vulgare *[36,37], *Triticum aestivum *[38,39], *Cajanus cajan *[40], *Sorghum bicolor *[41] and many others. DArT markers were also employed in the construction of a physical map of wheat chromosome 3B [42].

In this study we report on the development of a DArT array for five agronomically important species within the *Festuca-Lolium *complex. We demonstrate the utility of the approach for the estimation of intra- and interspecific genetic diversity. Moreover, by combining the DArT array with cytogenetic information in specific stocks of Festulolium, we illustrate successful anchoring of sets of DArT markers to individual chromosomes and chromosome bins.

## Results

### Development of DArT array

We developed a DArT array containing 7680 probes derived from methyl-filtered (through the use of *Pst*I restriction enzyme) genomic representations. In the first marker discovery experiment performed with 40 genotypes from each of the species *L. perenne*, *L. multiflorum*, *F. pratensis *and *F. arundinacea*, and seven genotypes of *F. glaucescens*, we identified 3884 polymorphic markers with standard DArTsoft settings. This is the highest frequency of polymorphism (50.6%) reported for DArT arrays, consistent with a very high level of DNA sequence variation in the fescue/ryegrass complex. Such a high degree of polymorphism was not unexpected, because the array consists of five different species of two genera. Most of these markers detected reliable polymorphisms across all accessions tested, while a subset of markers worked reliably only in a subset of diversity tested on the array.

In order to estimate the level of redundancy of markers on the array we used 184 festuloliums samples. It is important not to confuse the apparent redundancy (very high level of correlation between pairs of markers) which is due to Linkage Disequilibrium (LD) in the material analysed and probe sequence redundancy due to sampling the same (or highly similar) restriction fragment due to the random cloning process. The festuoliums lines we used for redundancy analysis were derived from various parents through interspecific hybridizations therefore they are expected to have very low level of LD. We applied correlation analysis of all possible pairs of 2352 markers discovered in the analysis (data not presented) and found a low level of redundancy: out of 1983 unique score signatures we found 1713 singletons, 198 bins with 2 markers, 53 bins with 3 markers, 11 bins with 4 markers, 6 bins with 5 markers and single bins with 6 and 7 markers, respectively. The detected level of redundancy (16%) will be verified in the future through sequencing, but taking this preliminary estimate we can conclude that there are approximately 3260 unique markers on the array (0.84 × 3884).

### Analysis of genetic diversity using DArT markers

Of the 3884 polymorphic markers identified on the array when polymorphic markers detected in each species were summed up only 2629 markers gave unequivocal scores in the joint fuzzy C-means clustering-based classification of all five species (see Additional file 1). Joint classification of signal on DArT arrays was compromised for the remaining 1255 markers by multiple alleles present in the same locus of the five species. Different alleles present in targets, especially when dealing with different species, often result in different levels of relative signal intensity for a probe on the array, either due to sequence or fragment length difference. These different levels of signal intensity do not conform to bimodal distribution DArTsoft requires for unambiguous 0/1 binarisation (scoring).

**Figure 1:**
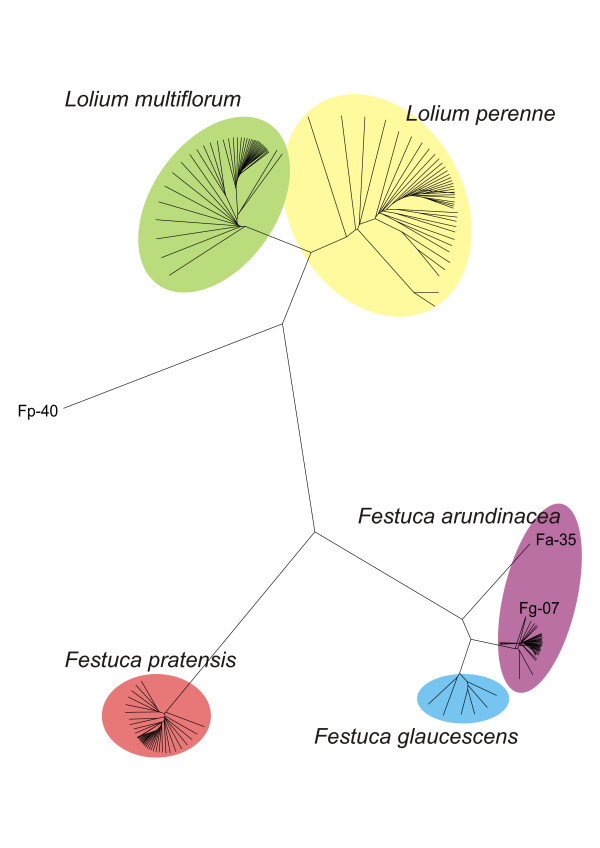
**UPGMA dendrogram (shown as Radial tree)**. UPGMA dendrogram (shown as Radial tree) based on hybridization of 80 *Lolium *and 87 *Festuca *genotypes to 2629 DArT markers and Felsenstein's modified Nei/Li restriction fragment distance. Two major groups representing the fescue and ryegrass genera are clearly differentiated. Both ryegrass species display higher genetic diversity than fescue species. Note that the accession of *F. arundinacea *Fa-35 (Moroccan ecotype 599533) was found distant of the major group. Similarly, one accession of *F. glaucescens *(Fg-7) clustered with the subgroup of *F. arundinacea*. Another inconsistent accession, *F. pratensis *Fp-40 (Norwegian cultivar Norild), was located separately outside of all other species in the dendrogram.

**Figure 2:**
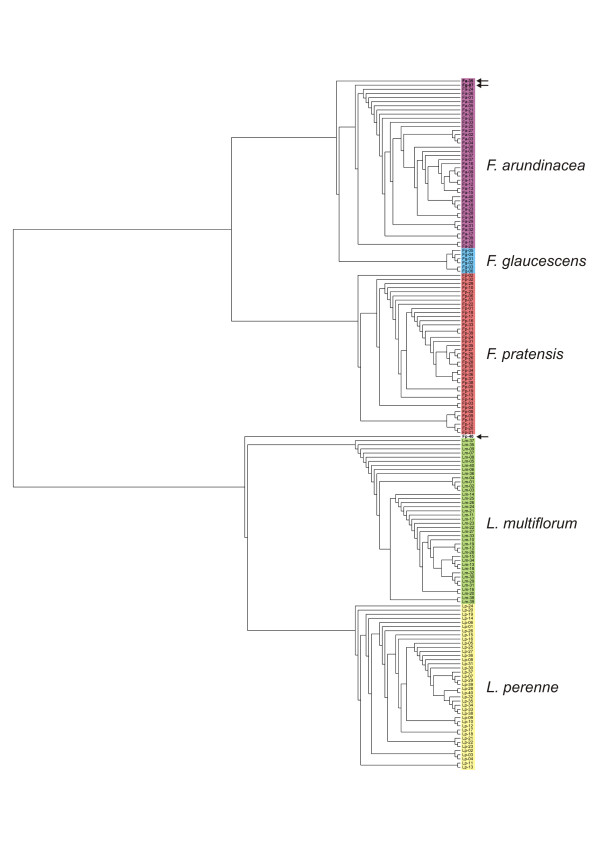
**UPGMA dendrogram (shown as Rectanglar cladogram)**. UPGMA dendrogram (shown as Rectanglar cladogram) based on hybridization of 80 *Lolium *and 87 *Festuca *genotypes to 2629 DArT markers and Felsenstein's modified Nei/Li restriction fragment distance. Groups of *Festuca *and *Lolium *accessions are marked using colored lines. Inconsistent accessions (Fp-40, Fg-7 and Fa-35) are marked using arrows.

Using these 2629 markers, we compiled a dendrogram including all 167 accessions of fescue and ryegrass. This differentiated two major groups, representing the fescue and ryegrass genera (Figures 1 and 2). Both ryegrass species analyzed (*L. perenne *and *L. multiflorum*) were closely related, but divergent enough to form separate groups. Fescue species formed two major groups. The first one included *F. pratensis *and, as the level of DArT polymorphism among *F. pratensis *accessions was low, they formed a tight group in the dendrogram. The second group included *F. arundinacea *and *F. glaucescens*. This group included two subgroups, one representing *F. arundinacea *accessions and the second *F. glaucescens *accessions. *F. arundinacea *accession Fa-35 (Moroccan ecotype 599533) was placed distant from the main *F. arundinacea *cluster. Similarly, one accession of *F. glaucescens *Fg-7 (genotype FRA001, obtained from Seed Bank, W. Reg. P. I. Station, Pullman, WA) clustered with the subgroup of *F. arundinacea*. All plants of this accession tested had chromosome numbers 2n = 42, indicating that this accession was probably a mislabeled *F. arundinacea*, and not of *F. glaucescens *origin. Moreover, the number of markers that scored positive ("1") in this accession (1253 markers) was higher than in any other accession of *F. glaucescens *(1059 - 1101 positive markers per accession) and was similar to those of *F. arundinacea *(1000 - 1351 markers per accession). Another inconsistent accession, *F. pratensis *Fp-40 (Norwegian cultivar Norild), was located outside of all other species in the dendrogram probably due to contamination of its DNA. Both accessions (Fg-7 and Fp-40) were excluded from further analyses.

The molecular genetic diversity analyses revealed several attributes characteristic for all species:

(1) Ecotypes from one country were usually more closely related than those from different countries (as in case of Lithuanian ecotypes of *F. pratensis *3982 and 3985).

(2) Cultivars released in one country usually displayed little polymorphism (as in case of *F. arundinacea *cultivars Eldorado and Wrangler released in USA and *L. perenne *cultivars Jakub and Kelt released in Czech Republic).

(3) Ryegrasses displayed higher levels of polymorphism than fescues.

### Polymorphism between the parents of mapping populations

With the prospect of using the array for mapping various agronomically important traits, we evaluated the presence of polymorphisms among (grand-) parents of mapping populations, which were deliberately included in our set of genotypes in the marker discovery stage of the project. It is clear that the level of marker polymorphism among the parents of mapping populations depends primarily on the purpose for which any specific map was developed. Where the purpose was to generate general genetic maps, the parents should be genetically as distant as possible, thus the number of polymorphic markers was expected to be high. This was the case for the *L. perenne *F2 mapping population VrnA, where 464 DArT markers were polymorphic between the grandparents NGB and VEYO (see Additional file 2). On the other hand, when a mapping population was generated to target a few specific traits, the parents can be genetically similar except for the region(s) harboring the gene(s) of interest. For example, only 289 polymorphic markers were found between LTS-01 and LTS-02. These two parental genotypes are both derived from similar gene pools, but designed to maximize heterozygosity for nitrogen use efficiency and cell wall digestibility, respectively.

### Genus- and species-specificity of DArT markers

**Figure 3:**
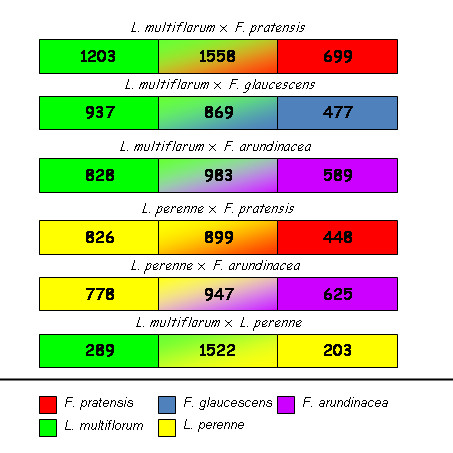
**DArT markers for hybrids**. Number of DArT markers which can be used to estimate the genomic constitution in hybrids within the *Festuca-Lolium *complex. In each pair-wise combination, a high proportion of markers is shared by both species. The markers shared by both parental genomes are in the middle section of the bar.

Reliable discrimination of DNA markers from both grass genera tested here, and possibly also from individual species, would greatly expand the utility of the DArT array. As the DArTFest array contained markers derived from both genera and all five species tested, our subsequent analysis focused on identifying genus- and species-specific markers. Of 3884 polymorphic DArT markers identified, over 1,000 markers detected a positive ("1") allele in each species (Table 1). The highest number of such markers "1" was in *F. pratensis *(between 1619 and 1821 markers per accession) and in *L. multiflorum *(between 1507 and 1852 markers per accession). Lower numbers of markers were detected in *F. arundinacea *(between 1000 and 1351 markers per accession), in *F. glaucescens *(between 1059 and 1101 markers per accession) and in *L. perenne *(between 821 and 1127 markers per accession). However, a large proportion of markers present in all five species tested reduced the numbers of species-specific markers. Thus, only nine species-specific markers were detected in *F. glaucescens*. For the two other fescue species, 34 and 123 species-specific markers were identified in *F. arundinacea *and *F, pratensis*, respectively. The number of markers shared between the allopolyploid *F. arundinacea *and its progenitors - *F. pratensis *and *F. glaucescens *- was high. Specifically, *F. arundinacea *shared 274 markers with only *F. glaucescens *and 82 markers exclusively with *F. pratensis*. In ryegrass, 52 markers specific for *L. perenne *and 82 markers specific for *L. multiflorum *were identified. Another 381 markers were present in both ryegrass species and absent in fescues. The number of markers, which could be used to identify particular genomes in intra- and interspecific hybrids, is shown in Figure 3.

**Table 1:** Species-specific DArT markers

**Species**	Scored markers*	Positive markers**	Polymorphic markers***	Species-specific markers****
*L. perenne*	2638	1725 (821-1127)	1407	52

*L. multiflorum*	3883	2761 (1507-1852)	2148	82

*F. pratensis*	3884	2257 (1619-1821)	1078	123

*F. glaucescens*	2630	1346 (1059-1101)	387	9

*F. arundinacea*	2638	1572 (1000-1351)	512	34

*) Note that some markers were not scored in all species

**) Range of positive markers for individual accessions is in parentheses

***) Markers polymorphic among the accessions

****) Identified after scoring 2629 markers

Number of species-specific DArT markers obtained from a DArT array containing 3884 polymorphic markers of 40 accessions each of *Lolium perenne *L., *L. multiflorum *Lam., *F. pratensis *Huds., *F. arundinacea *Schreb. and seven available accessions of *F. glaucescens *Boiss.

Based on the proportion of markers shared by pairs of species, genome relationships within this complex are as follow: Fa-Fg >> Lm-Lp >> Fa-Fp >> Fp-Lm > Fp-Fg > Fg-Lm > Fa-Lm > Fa-Lp > Fg-Lp > Fp-Lp. This correlates with the results of the cluster analysis.

### Mapping DArT markers to *F. pratensis *chromosomes and chromosome bins

We used a complete set of *Festuca-Lolium *single chromosome substitution lines of *F. pratensis *into tetraploid *L. multiflorum *to allocate DArT markers showing interspecific polymorphism to individual chromosomes of *F. pratensis*. In total, 160 DArT markers were anchored this way with six to 34 DArT markers anchored to a specific chromosome (Table 2). This represents 56% of all markers present in *F. pratensis *but absent in *L. multiflorum*. The lowest numbers of chromosome-specific markers were found on chromosome 4 (10 markers) and chromosome 7 (6 markers). However, another 18 markers were shared by the two chromosomes. This was surprising, as any other pair of chromosomes shared up to four markers only.

**Table 2:** DarT markers specific for chromosomes of *F. pratensis*

**Chromosome number** **(in Triticeae numbering system)**	**Number of chromosome-specific markers**
1	31

2	34

3	29

4	10

5	20

6	30

7	6

Number of the DArT markers specific for individual chromosomes of *F. pratensis*

**Figure 4:**
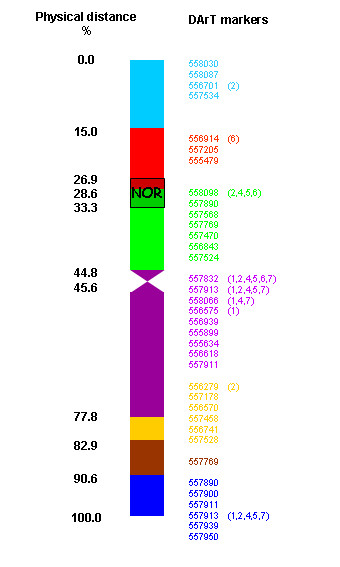
**DArT markers physically mapped to bins of *F. pratensis *chromosome 3**. Nine out of 36 markers co-localized to one or more chromosomes (chromosome numbers in brackets).

Based on the analysis of 14 recombinant lines with different lengths of the introgressed *F. pratensis *segments of chromosome 3, we were able to anchor 36 DArT markers to seven bins of this chromosome (Figure 4). Each bin contained between one and nine markers; nine markers were also shared with other chromosomes. The co-localization was found with all six remaining chromosomes with similar frequency.

## Discussion

The results of this study represent a major step toward the development of a high-throughput genotyping platform for important grass species from the genera *Festuca *and *Lolium*. Both genera contain agronomically important species, and are frequently intercrossed to combine complementary species characteristics. The 3884 DArT markers on the array is by far the highest number of markers ever used in studies of diversity or for genetic mapping of *Lolium *and *Festuca *species [7,16]. Our choice of the DArT array was motivated by the relative ease of its development, a good chance for extensive marker polymorphism, and its low cost per data point for future applications.

The results obtained in a set of 167 accessions representing two genera and five species proved the usefulness of the array to analyze genetic diversity. Intraspecific variability evaluated based on the detection of 2629 DArT markers was found to be higher in both ryegrass species as compared to the fescues. The lowest intraspecific variability was detected among the accessions of *F. arundinacea*. Using RAPD markers, Kölliker et al. [7] revealed much lower intraspecific variability in *F. pratensis *compared to *L. perenne*. Fjellheim et al. [43] found that the number of cpDNA haplotypes in *F. pratensis *was only about 25% of that observed in *L. perenne *by Balfourier et al. [44], and suggested that *F. pratensis *in Europe has been through a recent bottleneck. The higher intraspecific variability in ryegrasses compared with that in fescues was also noted based on screening of EST-SSR markers [9] and based on the observations of phenotypic traits (Černoch et al., unpublished). Although it was outside of the immediate goal of this study, the bottleneck created by interspecific hybridization in the evolution of tall fescues seems a reasonable assumption.

The set of genotypes used in this study included parents from the B14/16 × HF2/7 mapping population of *F. pratensis *[28]. The genetic map of this population contains 550 loci from homologous and heterologous RFLP, AFLP, isozyme, and SSR markers, and has a total length of 658.8 cM with an average marker density of 1.4 cM/marker. The number of polymorphic DArT markers between the parents of this mapping was 322. Thus, the number of DArT markers released in this study could significantly increase the density of the genetic linkage map.

Prior to this study, no high-throughput approach was available to study genomic constitutions of interspecific and intergeneric hybrids of *Festuca *and *Lolium*, nor in any other combination of frequently mated species/genera, such as wheat and rye. Yet, such systems would enable characterization of unknown accessions as well as hybrid breeding materials and cultivars, and assist greatly in early characterization of all primary recombinants that have to be generated in large numbers whenever high precision of alien introgression is required [45]. Our analyses indicate that the DArT array is very useful for these purposes. For each of the analyzed species, a number of species-specific markers were identified. For two species of high interest in relation to production of Festulolium cultivars, *L. multiflorum *and *F. pratensis*, over 400 species-specific markers were identified. Such a high number of markers will facilitate detailed analyses of genomic constitution in wide hybrids. It should also be possible to establish genome profiles specific for individual genotypes (e.g., cultivars), which could be useful in terms of legal protection of commercial cultivars.

In our previous work, we have shown that several hybrid Festulolium cultivars do not contain complete parental chromosome sets [31]. The range of applications of the DArTFest array to characterize genomic constitution in these cultivars would be greatly expanded, if DArT markers were assigned to individual chromosomes. This was partially achieved in this study using *Festuca-Lolium *chromosome substitution lines and 160 markers were anchored to individual chromosomes of *F. pratensis*. The observation of 18 markers co-localizing on chromosomes 4 and 7 could be explained by ancestral or more recent duplication(s).

In addition to varying proportions of parental chromosome sets, *Festuca × Lolium *hybrids often contain recombined chromosomes resulting from meiotic crossing-over between homoeologous chromosomes [31,46]. To describe genomes of hybrids at the subchromosomal level using DArT markers, these need to be assigned to specific chromosome regions via genetic mapping. However, as genetic linkage between genetic markers does not correspond to physical distances [47], physical mapping seems a better choice. In this study, we assigned a set of DArT markers to seven bins on chromosome 3 using 14 recombination lines of this chromosome. We developed about 30 such recombinant lines for each chromosome of *F. pratensis *and thus it will be possible to dissect the entire genome of *F. pratensis *into ca. 70 chromosomal bins. In a similar study, King et al. [47,48] utilized a set of introgression lines of diploid *L. perenne *harboring chromosome segments of *F. pratensis*. The authors reported the establishment of 18 bins for chromosome 3 of *F. pratensis *and physical localization of 104 AFLP markers. As DArT markers are much more amenable to multiplexing than AFLP markers (thousands rather than dozens of markers per assay) the throughput and cost advantage of the DArT array is significant (below 1 cent per datapoint). This advantage can be further enhanced through planned array expansion that could easily double the number of markers per assay, thereby further reducing the cost per datapoint with a minimum increase in price per sample. In addition, DArT markers are "sequence-ready", cloned genomic fragments, which offer important advantages over AFLPs for physical and genetic mapping. Sequencing of DArT markers identified in this study will enable *in-silico *comparisons of physical maps of fescue and ryegrass chromosomes with the sequenced grass genomes.

## Conclusion

The results of this study suggest that the newly developed DArTFest array will find numerous applications in grass genetics and breeding. It can be used to characterize genetic diversity as well as to develop genetic maps and identify markers linked with traits of interest. The ability to physically map DArT marker using chromosome substitution and recombinant lines will support the development of integrated genetic and physical maps of fescue and ryegrass. We also envisage a great impact of the array on characterizing genomic constitution of interspecific an intergeneric hybrids at the chromosomal and subchromosomal level. This progress should provide tools to understand the behavior of hybrid genomes as well as to improve the breeding of grass cultivars.

The array data have been deposited on a publically available website . The website will be permanent and will be expanded by genetic and physical mapping data as they become available.

## Methods

### Plant material

For the development of the DArT array, 40 accessions each of *L. perenne *L. (2n = 2x = 14), *L. multiflorum *Lam. (2n = 2x = 14 and 2n = 4x = 28), *F. pratensis *Huds. (2n = 2x = 14) and *F. arundinacea *Schreb. (2n = 6x = 42, plus all seven available accessions of *F. glaucescens *Boiss. (2n = 4x = 28) were chosen to discover the maximum genetic variability within the *Lolium-Festuca *complex and included ecotypes, cultivars and parents of mapping populations (see Additional file 1).

To map DArT markers to individual chromosomes of *F. pratensis*, we used single chromosome monosomic and disomic substitutions of *Festuca *into tetraploid *L. multiflorum*, as described by Kopecký et al. [46]. Whenever possible, five plants of each substitution line were used. To test whether DArT markers could be anchored to defined chromosome intervals, we selected 14 recombinant lines of *F. pratensis *chromosome 3 (using the *Triticeae *chromosome nomenclature system) in tetraploid *L. multiflorum*, with various lengths of *Festuca *chromatin present. These recombinants were selected from among backcross progeny of a monosomic substitution of chromosome 3 in tetraploid *L. multiflorum *(unpublished data).

### Development of the DArTFest array

For each of the five species tested, we developed a library of DArT clones using the *PstI/TaqI *method of complexity reduction. Both methods of complexity reduction and procedures for the library construction were performed as reported by Akbari et al. (2006). Each library consisted of 1536 clones organized into 4 microtiter plates with 384 clones per plate. Inserts from individual clones were amplified in the 384 microtiter plates using M13 primers so that part of the polylinker region of the cloning vector was co-amplified [34]. The amplicons were dried at 37°C, washed with 70% ethanol, and dissolved in a spotting buffer developed specifically for the Erie Scientific poly-L-lysine microarray slides (Wenzl *et al*., in preparation). The arrays containing inserts from 7680 clones were printed in duplicate using a MicroGridII arrayer (Biorobotics, Cambridge, UK) onto poly-L-lysine-coated slides (Erie Scientific, Portsmouth, NH, USA). Arrays were hybridized with fluorescently labeled targets from all genotypes used for the array development. Targets were prepared using a *PstI/TaqI *complexity reduction method [38]. For the marker discovery experiment, two replicated targets were analyzed for each genotype so that the technical reproducibility of markers discovered could be easily evaluated by comparing scoring consistency among technical replicates.

After overnight hybridization at 62°C, the slides were washed and scanned using a Tecan LS300 (Grödig, Salzburg, Austria) confocal laser scanner. Three images were generated from each slide: the image produced with a 488 nm laser was used for quality control and image processing was measuring the intensity of hybridisation of the reference (vector's polylinker) labelled with FAM fluorescent dye, and two images representing two independent targets, one produced with the 543 nm laser (Cy3 labelled targets) and one produced with the 633 nm laser (Cy5 labelled targets). The image processing and marker classification were performed using DArTsoft version 7.3 (DArT Pl, unpublished), a dedicated software package developed at DArT P/L (Yarralumla, Australia). Briefly, the relative hybridisation intensity of each clone on each slide was determined by dividing the hybridisation signal in the target channel (genomic representation) by the hybridisation signal in the reference channel (polylinker). Clones with variable relative hybridisation intensities across slides were subjected to fuzzy k-means clustering to convert relative hybridisation intensities into binary scores (presence *vs*. absence). The clustering was performed on averaged log-transformed relative intensity of two replicates printed for each probe on the array. The markers reported in this paper were selected with a call rate >80% and with technical reproducibility of above 99%.

We processed individual samples in the same way as samples for marker discovery experiments using similar marker quality thresholds in DArTsoft analysis.

### Analysis of genetic diversity

The DArTsoft-generated 0-1 scores were used as input for the RESTDIST and NEIGHBOR programs of the PHYLIP 3.6 software package to construct a dendrogram based on the Unweighted Pair Group Method with Algorithmic Mean (UPGMA) and Felsenstein's modification of the Nei/Li restriction fragment distance [49,50].

### Mapping DArT markers to fescue chromosomes and chromosome bins

To anchor markers to individual chromosomes of *F. pratensis*, DNA isolated from monosomic and disomic substitution lines for individual chromosomes of *F. pratensis *in tetraploid *L. multiflorum *were hybridized to the DArT array. A marker present in *F. pratensis *and absent in *L. multiflorum *was assigned to a chromosome if it was present in at least one substitution line for a particular chromosome. Several markers were assigned to multiple chromosomes. The same approach was used to anchor markers allocated on bins of *F. pratensis *chromosome 3 using the recombinant lines described above.

## List of abbreviations used

AFLP: amplified fragment length polymorphism; CAPS: cleaved amplified polymorphic sequence; cpDNA: chloroplast DNA; DArT: Diversity Arrays Technology; DArTFest: DArT array for the *Festuca*: *Lolium *complex; EST: expressed sequence tag; Fa: *Festuca arundinacea*; Fg: *Festuca glaucescens*; Fp: *Festuca pratensis*; GISH: genomic *in situ *hybridization; ITS: internal transcribed spacer; Lm: *Lolium multiflorum*; Lp: *Lolium perenne*; MAS: marker assisted selection; Mb: megabase; QTL: quantitative trait loci; RAPD: random amplified polymorphic DNA; RFLP: restriction fragment length polymorphism; SFP: single feature polymorphism; SNP: single nucleotide polymorphism; SSR: simple sequence repeat.

## Competing interests

AK (Director), HB and VC are affiliated with Diversity Arrays Technology Pty Ltd who provide DArT array commercial genotyping services for a range of crops.

## Authors' contributions

DK, JB, AJL, JHB, VC, RK, OAR and BS participated in the collection of plant material. AJL and DK developed the substitution and recombinant lines and characterized them cytologically. DK and JB extracted DNAs and checked the quality. The array was developed by HB, VC and AK. The data analysis was performed by DK, JB and AK. PS created a public website for data deposition. TL and JD made an intellectual contribution to the concept of the experiment. DK drafted the manuscript. AK, JD and AJL revised manuscript critically for important intellectual content. All authors read and approved the final manuscript.

## Supplementary Material

Additional file 1 **Hybridization scores**. The data provided represent scores obtained after hybridization of *Festuca *and *Lolium *genomic DNAs on the DArTFest chip. Click here for file

Additional file 2 **List of accessions**. The list provides the description of all accessions used for the development of the DArTFest chip. Click here for file
